# Identification of Imitation Cheese and Imitation Ice Cream Based on Vegetable Fat Using NMR Spectroscopy and Chemometrics

**DOI:** 10.1155/2013/367841

**Published:** 2013-06-06

**Authors:** Yulia B. Monakhova, Rolf Godelmann, Claudia Andlauer, Thomas Kuballa, Dirk W. Lachenmeier

**Affiliations:** ^1^Chemisches und Veterinäruntersuchungsamt (CVUA) Karlsruhe, Weissenburger Strasse 3, 76187 Karlsruhe, Germany; ^2^Department of Chemistry, Saratov State University, Astrakhanskaya Street 83, 410012 Saratov, Russia; ^3^Bruker Biospin GmbH, Silbersteifen, 76287 Rheinstetten, Germany; ^4^Ministry of Rural Affairs and Consumer Protection, Kernerplatz 10, 70182 Stuttgart, Germany

## Abstract

Vegetable oils and fats may be used as cheap substitutes for milk fat to manufacture imitation cheese or imitation ice cream. In this study, 400 MHz nuclear magnetic resonance (NMR) spectroscopy of the fat fraction of the products was used in the context of food surveillance to validate the labeling of milk-based products. For sample preparation, the fat was extracted using an automated Weibull-Stoldt methodology. Using principal component analysis (PCA), imitation products can be easily detected. In both cheese and ice cream, a differentiation according to the type of raw material (milk fat and vegetable fat) was possible. The loadings plot shows that imitation products were distinguishable by differences in their fatty acid ratios. Furthermore, a differentiation of several types of cheese (Edamer, Gouda, Emmentaler, and Feta) was possible. Quantitative data regarding the composition of the investigated products can also be predicted from the same spectra using partial least squares (PLS) regression. The models obtained for 13 compounds in cheese (*R* ^2^ 0.75–0.95) and 17 compounds in ice cream (*R* ^2^ 0.83–0.99) (e.g., fatty acids and esters) were suitable for a screening analysis. NMR spectroscopy was judged as suitable for the routine analysis of dairy products based on milk or on vegetable fat substitutes.

## 1. Introduction 

Due to industry efforts to provide low-cost foods or due to general ethical considerations against cow's milk consumption [1], imitation dairy products have recently appeared on the market [2–5]. Cheese analogues or imitation cheese are cheese-like products in which milk fat, milk protein, or both are partially or completely replaced with nonmilk-based components such as soy [2], starch [6], or vegetable replacers [3]. Other alternative products for consumers with cow milk intolerance [7] based on goat [8, 9] or sheep milk [9] can also be found on the market. Vegetable oils and fats are most commonly used as cheap substitutes for milk fat to manufacture imitation cheese or imitation ice cream. While not being harmful to health, the imitation products may be of lesser nutritional quality (e.g., by lower calcium content) and contain several artificial flavors and food colors [10]. Unfortunately, such imitation products may be offered without the necessary labeling, which is a deception of the consumer. Pizza topping is a good example of such a possibility [10]. It has therefore become necessary to develop a reliable technique able to detect such products in the market.

Chromatographic methods are the most popular choice for analysis of organic substances in cheese. For example, gas chromatography (GC) with mass-spectrometric detection [11–13] or flame ionization detection [11] and high-performance liquid chromatography [9, 13, 14] were previously applied. With these methods, precise and diverse information about volatile profiles of the particular type of cheese could be obtained. This has been done, for example, for Reggianito Argentino cheese [11], Italian mountain cheese (Bitto) [15], Majorcan cheese [16], Kuflu Turkish cheese [13], and different varieties of goat and sheep cheese [9, 12]. However, due to the matrix complexity of dairy products chromatographic analysis usually involves pretreatment steps such as solid-phase extraction (SPE) [17] or headspace sorptive extraction (HSSE) combined with thermal desorption (TD) [15]. Therefore, it can be concluded that chromatographic techniques are accurate but laborious, expensive, and time consuming.

Other methods based on spectroscopic techniques are also available. These include Fourier transform infrared (FTIR) spectroscopy [18–20], visible-near infrared reflectance spectroscopy [21], near infrared (NIR) spectroscopy [20, 22], atomic absorption spectroscopy [23], inductively coupled plasma optical emission spectrometry [24, 25], and fluorescence spectroscopy [20]. Fluorescence spectroscopy is the most sensitive method but only few compounds give rise to fluorescence signals. In FTIR and NIR spectra, strong and broad signals of water prevent the informative characterization of dairy products.

Among spectroscopic techniques in the area of food analysis, NMR is currently on the rise [26]. Previous application areas include beer [27], juice [28], grapes [29], infant formulas, [30] or pine nuts [31]. The application of NMR spectroscopy to cheese and ice cream analysis has been also presented in several studies. ^1^H NMR was used to investigate the influence of packaging on the degradation of soft cheese [32]. Full ^1^H NMR assignments of signals of the water fractions of different types of cheese were recently provided [25, 33, 34]. ^1^H NMR is also able to provide reliable qualitative and quantitative analysis of amino acids [35] and biogenic amines [36] in cheese. ^31^P and ^23^Na NMR were used for the investigation of both phosphate and sodium ion distribution in semihard cheese [37]. A time-domain nuclear magnetic resonance (TD-NMR) was applied to the quick determination of moisture profiles during cheese drying [38]. Regarding NMR analysis of ice cream, TD-NMR was previously used for the investigation of the aggregation state (liquid or solid) of water and fat [39–41]. Another article utilized site-specific natural isotope fractionation NMR to detect adulteration of vanillin in ice cream [42].

Despite the mentioned diverse studies about composition of dairy products, there are only few articles dealing with the detection of their adulteration. For example, it was demonstrated that it is possible to identify the presence of cow milk in buffalo mozzarella by the use of electrophoretic mobility of cow and buffalo casein [43]. A method based on triacylglycerol composition obtained with GC-FID of cheese was also proposed to detect the levels of foreign fat [44]. Other techniques based on the determination of particular markers were also reviewed [4]. All of them are based on time-consuming chromatographic measurements and, what is more important, are able to detect only specific types of adulteration. 

In the view of these facts, NMR seems to be promising to provide accurate classification of dairy products according to the raw material origin. Therefore, the main objective of this research was to investigate the ability of NMR spectroscopy to differentiate milk fat products from vegetable fat substitutes. Cheese and ice cream were chosen as examples.

## 2. Experimental Section

### 2.1. Samples

A total of 109 cheese samples and 112 ice cream samples based on milk fat were analyzed. The products were either purchased at local stores in Karlsruhe, Germany, or submitted to the CVUA Karlsruhe for official food control purposes in Baden-Württemberg, Germany. Samples were selected to cover all possible imitation products available on the German market and a wide composition variability of milk fat products. Furthermore, imitation products based on vegetable fat (or vegetable fat/milk fat mixture) were analyzed (*n* = 11 cheese and *n* = 11 ice cream). All samples were subjected to the standard GC/MS analysis that confirmed the labeling information in every case.

### 2.2. Sample Preparation and Validation

**Figure 1:**
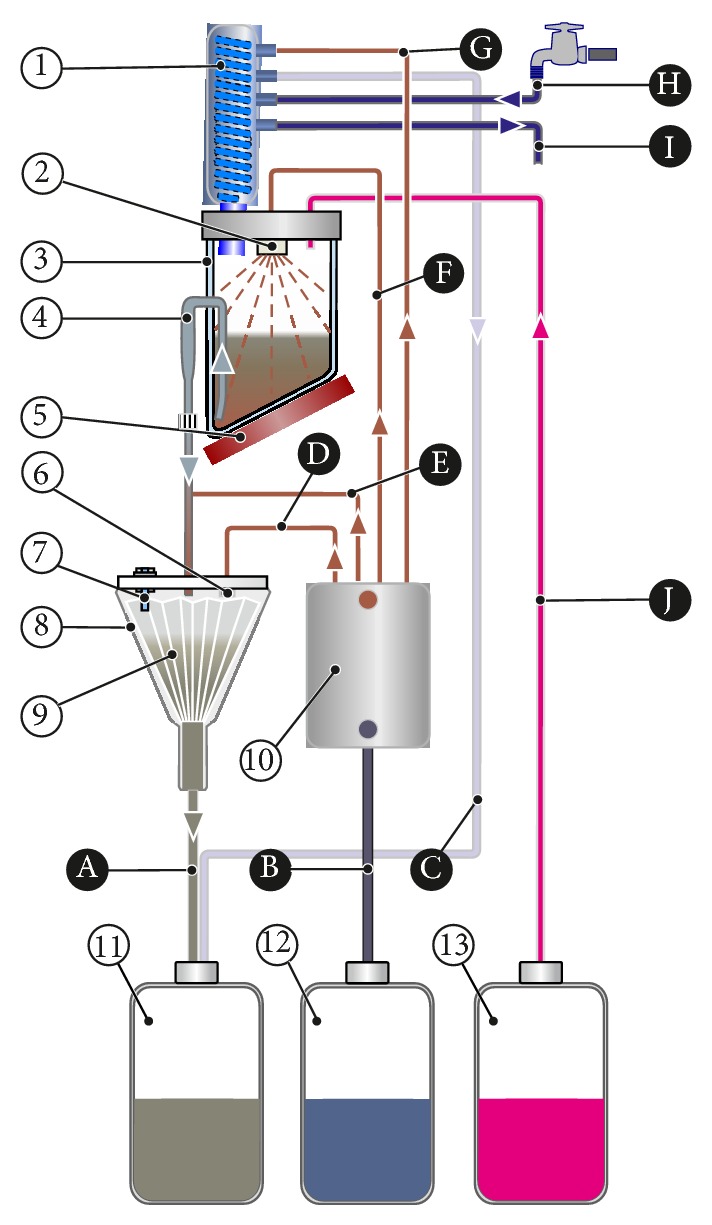
Schematic illustration of the automatic sample hydrolysis process necessary for Weibull-Stoldt fat extraction (reproduced with permission from Gerhardt Analytical Systems). 1 Condenser, 2 shower, 3 hydrolysis beaker, 4 sample transfer device, 5 hotplate, 6 shower for filter, 7 level sensor funnel, 8 funnel, 9 folded filter, 10 hot water generator, 11 tank for sample waste, 12 tank for H_2_O, and 13 tank for HCl, A sample drainage, B distilled water addition, C air ventilation for condenser, D hot water addition-filter moisture, E hot water addition-rinsing sample transfer, F hot water addition-rinsing hydrolysis beaker, G hot water addition-rinsing condenser, H cooling water inlet, I cooling water outlet, J hydrochloric acid addition.

Sample preparation of cheese and ice cream was conducted by the German reference Weibull-Stoldt methodology for fat hydrolysis and extraction. The hydrolysis of the sample was conducted using the automated hydrolysis system HYDROTHERM (Gerhardt Analytical Systems, Königswinter, Germany) as shown in Figure 1. Briefly, a representative average sample (at least 200 g) is minced and homogenized. Then 10 g of the homogenized sample is weighed and put into the digestion beaker for automated hydrochloric acid hydrolysis. After the addition of hydrochloric acid (4 mol/L, 150 mL), the liquid is then quickly brought to boil and simmered for about 1 hour. At the end of hydrolysis the digestion mixture is diluted with hot water (100 mL) to the double amount and then is immediately filtered through pleated filter, which has been moistened automatically by the system with water (number of moisture cycles = 3 and water amount per cycle = 40 mL). After the program has finished, the filter is placed on a watch glass and dried for up to 1.5 h at 103 ± 2°C in a drying oven. After cooling off, the fat is extracted using Soxhlet extraction with petroleum ether.

After finishing, the extraction flasks are dried in the drying oven for 60 minutes at 103 ± 2°C. Then, they are placed in a desiccator, left to cool down to room temperature. After a constant weight was achieved, the fat phase was ready for NMR analysis, for which 200 mg of the fat fraction is mixed with 0.80 mL of CDCl_3_ containing 0.1% tetramethylsilane (TMS). 0.6 mL of the mixture is poured into an NMR tube and directly measured.

To investigate the reproducibility of the sample preparation, two different imitation cheese samples were prepared twice and several resonances were integrated: 9.76–9.74 ppm (triplet), 4.33–4.30 ppm (doublet), and 2.80–2.72 ppm (triplet). The reproducibility was then calculated as relative standard deviation (RSD) between replicates.

### 2.3. NMR Measurements at 400 MHz

All NMR measurements were performed on a Bruker Avance 400 Ultrashield spectrometer (Bruker Biospin, Rheinstetten, Germany) equipped with a 5 mm SEI probe with Z-gradient coils, using a Bruker Automatic Sample Changer (B-ACS 120). All spectra were acquired at 300.0 K. The data were acquired automatically under the control of ICON-NMR (Bruker Biospin, Rheinstetten, Germany), requiring about 12 min (^1^H NMR) and 30 min (^13^C NMR) per sample. All NMR spectra were phased and baseline corrected.

### 2.4. NMR Spectra Acquisition

^1^H NMR spectra were acquired using the Bruker 1D zg pulse sequence with 128 scans (NS) and 2 prior dummy scans (DS). The sweep width (SW) was 20.5503 ppm and the time domain (TD) of the free induction decays (FIDs) was 131 k, acquisition time (AQ) was 7.97 s, and the repetition time (D1) was 1.0 s. Receiver gain (RG) value was set to 8.0. For acquisition of  ^13^C NMR spectra, the Bruker pulse sequence zgpg was used. After the application of 4 DS, 8 FIDs (NS = 1024) were collected into a TD of 131072 (131 k) complex data points using a 238.8728 ppm SW and a RG of 2050 (AQ = 1.38 s and D1 = 2.00 s). 

### 2.5. Nontargeted Analysis and Chemometrics

The resulting spectra were analyzed using the software Unscrambler X version 10.0.1 (Camo Software AS, Oslo, Norway). We tested several spectral regions for calculation: aliphatic (0.25–3 ppm), midfield (3–6 ppm), aromatic (6–10 ppm) as well as the 0.25–6 ppm region with 0.01 ppm bucket width. Details on the bucketing process of NMR spectra for multivariate data analysis were previously described [27].

The technique of cross-validation was applied to determine the number of principal components (PCs) needed. For cheese spectra differentiation, we used 7 PCs (explained variance 97%) for ^1^H NMR and 8 PCs (explained variance 98%) for ^13^C NMR. The PCA model for ice cream spectra required 8 PCs (explained variance 97%). Using PLS regression, the NMR spectra were correlated with reference GC analysis data. PCA and PLS models were validated via full cross-validation. Furthermore, the PLS models were evaluated via test set validation (*n* = 10), and results are compared with those obtained from a standard GC method.

## 3. Results and Discussion

### 3.1. Sample Preparation and Spectra Analysis

Cheese and ice cream cannot be directly measured with liquid-state NMR, so that a sample preparation has to occur aiming to provide a measurable liquid solution. According to the literature, the most meaningful information about discrimination between types of cheese is contained in the fat fraction [45–47]. For this reason, we decided to apply the fat obtained with the Weibull-Stoldt methodology for our NMR analysis. According to Weibull-Stoldt, the sample is first hydrolyzed to free the fat, and then the fat fraction was extracted from the rest of the sample using a solvent. The use of the Weibull-Stoldt fat had also the advantage that this methodology is already conducted for nearly all samples of cheese and ice cream that reach our laboratory, as the labeled fat content on the package has to be controlled. The Weibull-Stoldt fat was also used for standard GC analysis. To simplify the Weibull-Stoldt protocol, we applied an automated device for the hydrolysis, which is the first system worldwide that was recently commercialized for this purpose. Prior to its application, we conducted this procedure using manual hydrolysis according to the Weibull-Stoldt method. The NMR spectra obtained with both methods showed the same fatty acid profile; however, the automated device was considerably more efficient as it is possible to prepare 6 samples at once without human intervention. 

To demonstrate the reproducibility of this method, replicate measurements of different samples were performed. The relative standard deviations (RSD) between the two measurements were found to range between 0.1% and 2.1% (9.76–9.74 ppm), 1.0% and 1.2% (4.33–4.30 ppm), and 0.7 and 3.1% (2.80–2.72 ppm) for imitation cheese samples. The data indicated that the sample preparation procedure is adequately reproducible to facilitate a comparison between different cheese and ice cream samples. 

**Figure 2:**
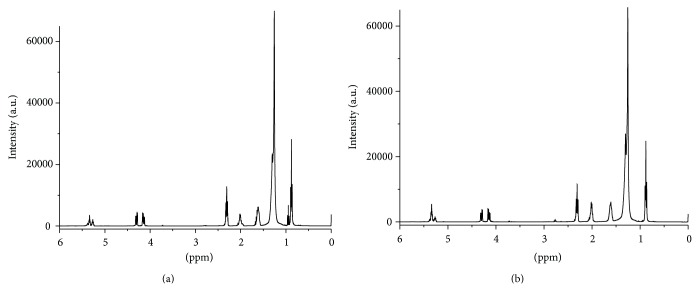
^1^H NMR spectra of Gouda cheese (a) compared to an imitation cheese based on vegetable fat (b).

Figure 2(a) showed the ^1^H NMR spectrum of a representative sample of Gouda cheese. The signal of triglycerides and fatty acids dominated the spectrum [46]. Imitation cheese displayed a similar fatty acid profile (Figure 2(b)). By inspection of these spectra, we found the differences in the intensity of resonances relative to methyl (1.00–0.90 ppm) and bis-allylic protons (5.00–4.90 ppm) between the two groups of the products. In the ^13^C NMR spectra, differences in the 173–170 ppm region (butyric acid) can also be observed. The same findings were valid for ice cream samples. Nevertheless, it can be concluded that NMR spectra of cheese and ice cream are very complex and a strong overlap of the resonances occurs. In the following, dairy products properties were uncovered from the NMR spectra using multivariate data analysis.

### 3.2. Nontargeted Analysis

The spectroscopic data were visualized either through PCA scatter plots, in which each point represents an individual sample, or through loadings plots, which permit the identification of the most important spectral regions to separate the clusters and, therefore, reveal markers (compounds that are responsible for differentiation). 

**Figure 3:**
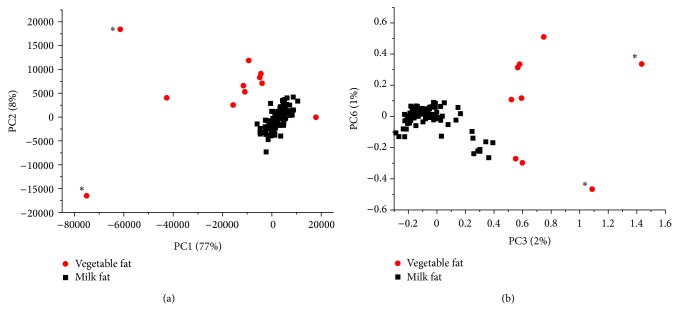
Scatter plot of the PCA scores for ^1^H NMR ((a), 3.0–0.25 ppm) and ^13^C NMR ((b), 200–0.25 ppm) for cheese samples (stars denote tzatziki samples).

At first, PCA was performed on NMR spectra of cheese samples. The best grouping of similar samples was observed in the PCA scores plots of PC1-PC2 (^1^H NMR spectra, Figure 3(a)) and PC3-PC6 (^13^C NMR spectra, Figure 3(b)). On both plots, the imitation cheese samples were clearly separated from all of the remaining ones and were clustered in the range of negative values of PC1 (^1^H NMR) or positive values of PC3 (^13^C NMR). Furthermore, on both plots two especially conspicuous imitation samples were observed (marked with stars on Figures 3(a) and 3(b)). These two products represented tzatziki (a traditional Greek appetizer), which consists of both milk fat with vegetable fat and olive oil addition. Additionally, one outlier was located in the positive values of PC1 (Figure 3(a)). In addition to vegetable fat, this sample contained also about 3% of milk fat (as proven by GC analysis). Cheese made from milk (cow, goat, and sheep) was clustered around 0 in both PCA scores plots. Overall, we think that PCA in the aliphatic ^1^H NMR region (Figure 3(a)) provided better differentiation of cheese samples. In this case, the samples in the imitation cluster were located closer to each other and the distance between milk fat/vegetable fat clusters was larger than that obtained with ^13^C NMR data. Furthermore, vegetable fat/milk fat mixtures can also be recognized with ^1^H NMR spectra.

**Figure 4:**
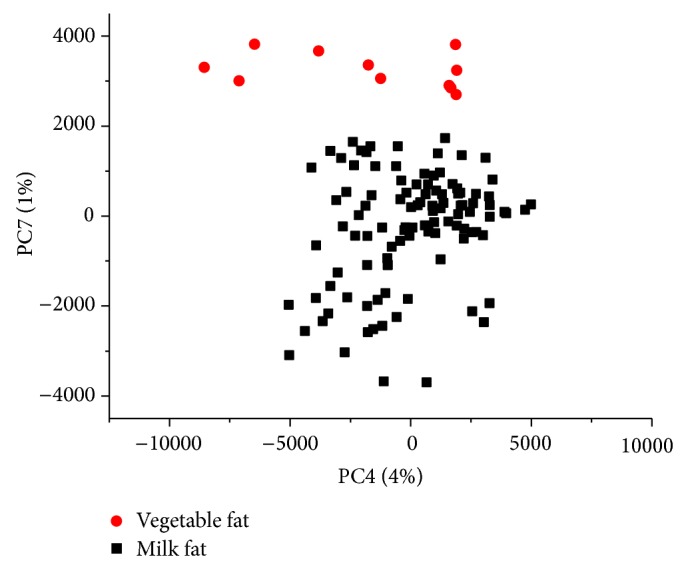
Scatter plot of the PCA ^1^H NMR scores in the 3.0–0.25 ppm region for ice cream samples.

The PCA scatter plot of ^1^H NMR spectra (3–0.25 ppm) of ice cream samples was shown in Figure 4. In this case, seven PCs were found sufficient for differentiation. Unlike cheese samples, for which a good discrimination was observed between the first two PCs (Figure 3(a)), for ice cream the best model was constructed between PC4 and PC7. For ice cream samples, therefore, the variability in minor compound concentrations (such as alcohols and long-chain fatty acids) influenced the discrimination.

The chemical shifts and the associated functional groups that were responsible for the differentiation of the dairy products can be identified using the loadings plots. In the loadings plot, each chemical shift was plotted against its importance in discriminating the samples. In our case, the spectral regions 1.00–0.90 and 5.00–4.90 ppm were found to be important in the milk fat/vegetable fat product differentiation (for both cheese and ice cream). These regions consisted of the signals from methyl groups of different compounds and olefinic protons of all unsaturated chains [46]. The buckets at 2.32 and 2.30 ppm (most probably methylenic protons bonded to C2 of all fatty acid chains) [46] were also important for ice cream products. 

Due to the low number of different imitation products currently available on the German market (probably because of a recent media campaign against these products [10]), we were not able to analyze our data with classification methods such soft independent modeling of class analogy (SIMCA) or linear discriminant analysis (LDA), which would require a larger dataset for training and validation. However, new samples can be distinguished by adding them to the developed PCA model.

**Figure 5:**
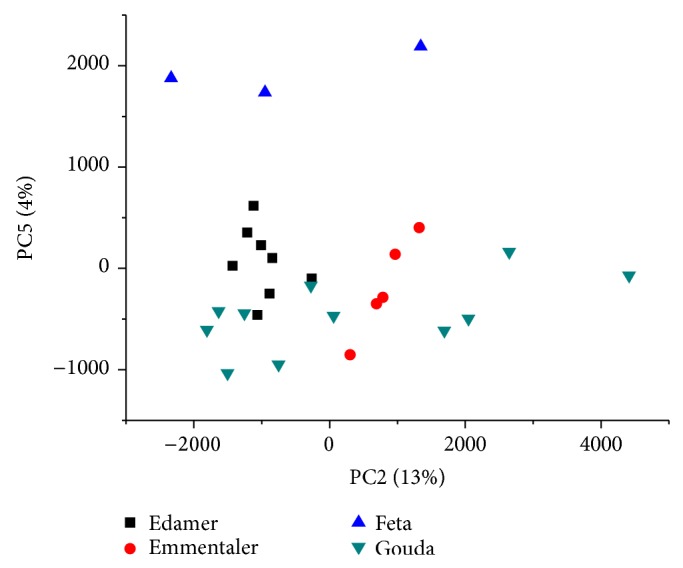
PCA of cheese types in the aliphatic region (3.0–0.25 ppm).

While the differences of fat material used for cheese manufacture can be seen within the first several PCs (Figure 3(a)), higher PCs could uncover further clustering. To do this, we removed the imitation products and repeated the PCA. It can seen from Figure 5 that grouping in respect to the cheese types (Edamer, Gouda, Feta, and Emmentaler) is observed. It is not surprising because every cheese type was produced differently and, therefore, had a unique fatty acid profile. The two separate Gouda clusters were separated probably due to different ripening times similar to what was previously found for Italian Parmigiano Reggiano cheese [34]. It should be noted that ^13^C NMR cannot provide such clear differentiation (only Emmentaler, Gouda, and Feta can be classified). 

A recent review discussed the potential of different techniques coupled with chemometric analysis for the determination of the quality and the authenticity of dairy products, from which NMR plays an important role [47]. In the study of Rodrigues et al. [33] it was shown that metabolic profiling obtained by NMR combined with multivariate analysis allows to distinguish cheese samples in terms of maturation time, as well as added probiotic and prebiotic substances. PCA analysis was also performed on ^1^H NMR spectra of Italian Parmigiano Reggiano cheese to control time of ripening [34]. In the same research, the authors were able to provide geographical differentiation of cheese samples with Partial Least Squares-Discriminant Analysis (PLS-DA). ^1^H and ^13^C NMR coupled with PCA was used to differentiate PDO Asiago cheese produced in different areas [46]. High-resolution magic angle spinning (HR MAS) NMR together with PCA was able to distinguish Emmental cheese samples according to geographical region [48]. It should be mentioned, however, that data sets in all of these studies involved not more than 30 samples and focused on one specific group or origin. Therefore, our investigation is the first to apply ^1^H NMR spectroscopy with multivariate methods to characterize a large number of commercial cheese and ice cream samples. Furthermore, to the best of our knowledge, no previous studies evaluated the performance of NMR spectroscopy to reveal vegetable fat adulteration of cheese and ice cream so far. 

### 3.3. Quantitative Prediction of Dairy Product Composition

Besides the qualitative classification of our samples, in order to perform quality control of dairy products, it is also necessary to quantify certain compounds (e.g., saturated and unsaturated fatty acids and their esters). GC is among the most common methods for determining the fatty acid composition of cheese and ice cream [9, 11, 12, 15, 17]. ^1^H NMR spectroscopic methods based on direct integration were also proposed for this purpose [45]. However, only a limited number of fatty acids can be quantified because they display similar and overlapping signals in the NMR spectra (Figure 2), which make simple quantification by integration of a distinct peak not possible. Moreover, in some cases the results can strongly deviate from the reference values [45]. 

To overcome these problems, we used Partial Least Squares regression (PLS) to correlate NMR spectra (in the 6–0.25 ppm range) to the data of reference GC analysis. Results (i.e., root mean square error (RMSE), correlation coefficient (*R* ^2^), the number of PLS factors as well as NMR range used) of the best-fitting PLS models for ice creams were listed in Table 1. Fourteen of seventeen models exhibited correlation coefficients greater than 0.90. The correlation coefficients for butyric acid (*R* = 0.89), octadecanoic acid (*R* = 0.83), and nonadecanoic acid (*R* = 0.89) also appeared to be adequate for a screening procedure. The separate PLS models constructed for cheese showed slightly lower but comparable performance (*R* ^2^ values were in the range of 0.75–0.95). Inadequate PLS models (*R* ^2^ < 0.50) were only obtained for pentadecanoic acid, margaric acid, octadecanoic acid, and nonadecanoic acid for cheese samples (in comparison to ice cream, Table 1) due to the small concentrations of these compounds. 

The PLS prediction models described previously were validated with an independent set of ten randomly chosen samples (only the acceptable models with *R* ^2^ > 0.90 were considered). The average relative deviations of the predicted values from the GC ones were in the range of 2–10% and 5–15% for ice cream and cheese samples. These data clearly demonstrated the reliability of the models and the potential of this technique for simultaneous quantitative analysis of dairy product characteristics along with the nontargeted control.

Therefore, we have extended quantitative NMR spectroscopy to 17 compounds that have to be analyzed during food control. In principle, NMR spectra contain the same information as GC but can be gathered much faster and more efficiently. The results emphasized the capability of NMR to rapidly and reliably predict cheese and ice cream characteristics.

## 4. Conclusions

Traditional analytical strategies to uncover adulteration of food rely on targeted analysis (in the course of which only certain marker compounds are analyzed). This approach has obstacles on many points, in particular, starting from time of analysis, use of sophisticated analytical equipment, or usage of expensive and/or toxic reagents. What is more important is that new forms of adulteration could not be uncovered as in the case of melamine adulteration of milk by applying the unspecific Kjeldahl assay [30]. A demand exists for rapid, accurate, and cheaper methods for direct quality measurement of food and food ingredients, which should include a nontargeted approach. 

Spectroscopic techniques combined with chemometric methods are a possible solution of this problem. From the wide range of spectroscopic methods, the high amount of spectral information of NMR is ideally suited for nontargeted analysis. This approach has only recently entered routine analysis in food control institutions. Examples are the investigation of infant formulas [30], pine nuts [31], or milk and milk substitutes [49]. This study has further shown that NMR is an efficient tool to detect fraud in the dairy product industry, protecting consumers against improper practices, and guaranteeing fair trade. 

The method of automated sample preparation (Weibull Stoldt hydrolysis) used in this paper appears to be extremely efficient, especially when dealing with a high number of samples to be analyzed using multivariate methods. This procedure allowed the collection of high-quality spectra with distinct spectral features that were consistent within each sample. The developed technique could be further applied to solve other classification problems (differentiation between milk fat from different animals besides cows, or geographic discrimination) and shows great promise as a rapid tool for cheese analysis.

## Figures and Tables

**Table 1:** PLS correlation between data of reference GC analysis and NMR spectra (6.0–0.25 ppm) for ice cream (*n* = 99).

Analytes	Reference range	PLS factors	Calibration		Validation	
			RMSE^a^	*R* ^2^	RMSE	*R* ^2^
Butyric acid (C4:0) (%)	0–25	7	0.21	0.89	0.24	0.85
Caproic acid (C6:0) (%)	0–1.9	7	0.15	0.87	0.17	0.84
Octanoic acid (C8:0) (%)	0–8.4	4	0.33	0.93	0.37	0.92
*n*-Capric acid (C10:0) (%)	0–6.7	4	0.28	0.91	0.33	0.87
Dodecanoic acid (C12:0) (%)	0–43.5	6	1.2	0.97	1.6	0.96
Tetradecanoic acid (C14:0) (%)	0–20.3	5	0.70	0.94	0.84	0.92
Myristoleic acid (C14:1) (%)	0–1.1	6	0.072	0.91	0.089	0.87
Hexadecanoic acid (C16:0) (%)	9.7–40.6	6	1.1	0.96	1.39	0.94
Palmitoleic acid (C16:1) (%)	0–1.7	6	0.09	0.94	0.11	0.91
Oleic acid (C18:1) (%)	5.2–60.7	4	1.0	0.99	1.5	0.97
Pentadecanoic acid (C15:0) (%)	0–1.6	7	0.099	0.93	0.12	0.90
Margaric acid (C17:0) (%)	0–0.8	7	0.04	0.92	0.05	0.89
Octadecanoic acid (C18:0) (%)	3.9–36	5	1.0	0.83	1.2	0.76
Nonadecanoic acid (C19:0) (%)	0–1.1	7	0.02	0.89	0.04	0.78
*cis*. *cis*-9.12-Octadecadienoic acid (C18:2) (%)	0.3–22.8	7	0.25	0.99	0.39	0.97
Methyl butanoate (g/100 g fat)	0–3.8	7	0.11	0.99	0.18	0.97
Hexanoic acid methyl ester (g/100 g fat)	0.04–2.24	7	0.07	0.98	0.09	0.96

^a^Root mean-squared error (RMSE) values are expressed in the same units as the analytes.
